# Subglottic Adenoid Cystic Carcinoma Mimicking Bronchial Asthma: A Case Report

**DOI:** 10.7759/cureus.60571

**Published:** 2024-05-18

**Authors:** K K Athish, Guruprasad T J, Spurthy Padmanabha, Harshitha K R

**Affiliations:** 1 Internal Medicine, Sri Devaraj Urs Academy of Higher Education and Research, Kolar, IND; 2 Respiratory Medicine, Sri Devaraj Urs Medical College, Kolar, IND; 3 Pulmonology, Sri Devaraj Urs Academy of Higher Education and Research, Kolar, IND

**Keywords:** bronchoscopy, fixed airway obstruction, laryngeal cancer, adenoid cystic carcinoma (acc), subglottic mass

## Abstract

Adenoid cystic carcinoma (ACC) is a rare type of tumor that usually originates from minor salivary glands in the oral cavity. ACC of the larynx is even rare. This case study describes a 36-year-old non-smoking male farmer who initially presented with dyspnea and was misdiagnosed with bronchial asthma. Spirometry revealed fixed airway obstruction. Further evaluation revealed a pedunculated mass obstructing the airway, which was diagnosed as ACC by histopathological examination of the biopsy specimen. The patient was treated with radiation therapy, resulting in clinical improvement after six weeks. ACC is highly invasive and slow-growing, with perineural extension and a higher risk of recurrence. Metastasis in the lungs is common. Adequate preoperative staging, including imaging with computed tomography (CT) and magnetic resonance imaging, is important for planning treatment. The role of radiation therapy with concurrent chemotherapy is still under trial.

## Introduction

Laryngeal carcinoma is the second most prevalent malignancy of the upper aero-digestive tract with over 13,000 cases documented yearly in the United States solely [1]. Despite squamous cell carcinoma (SCC) contributing to the majority (~90%) of malignant laryngeal tumors, the larynx is susceptible to a wide range of malignancies with different histologic characteristics [2]. ACC is a relatively slow-growing tumor that most frequently arises from minor salivary glands of the oral cavity, paranasal sinuses, pharynx, and larynx [3]. Adenoid cystic carcinomas (ACC) predominantly affect salivary glands of the oral cavity. ACC of the larynx is one of the rarest variants accounting for <1% of laryngeal tumors, originating along the mucosa of the laryngo-tracheal tract, as there are few salivary glands [4,5]. The available research estimates that there have only been less than 100 case reports on ACC published globally [6]. In contrast to SCC, ACC of the larynx is not related to a history of smoking or no distinct predisposing risk factors to this malignancy [4,5]. Hoarseness and dyspnea are frequently the predominant symptoms, while local-regional metastases are extremely rare, at least for cancers that start in the subglottic region [6,7]. The median survival period is about eight years [7]. Although the clinical behavior of extra laryngeal ACC is well known, currently only limited case series are available for laryngeal ACCs. Here, we describe a case of ACC of the larynx with an atypical presentation that was initially misdiagnosed as asthma. However, the following discussion details how further evaluation helped in arriving at an accurate diagnosis.

## Case presentation

A 36-year-old Indian farmer, a non-smoker male, presented with a history of dyspnea for five months, which aggravated on exertion, for which he was treated for bronchial asthma with bronchodilators in a local hospital. His dyspnea had worsened for half a month and was associated with noisy breathing for five days. The patient also complained of a cough for one week associated with yellow mucoid, non-foul smelling, and occasionally blood-tinged sputum. He complained of hoarseness of voice for five days. He denied a history of chest pain, palpitations, orthopnea, facial puffiness, upper limb swelling, difficulty in swallowing, fever, loss of weight, and loss of appetite. The patient had no known comorbidities. He gave the history of a road traffic accident 12 years ago with blunt trauma chest and rib fractures.

Physical examination on presentation revealed a temperature of 36.9°C, pulse of 91 bpm, blood pressure of 124/80 mmHg, respiratory rate of 23 cycles/minute, and oxygen saturation of 96% on room air. Stidor was present. Respiratory system examination revealed no abnormality on inspection, palpation, and percussion. Bilateral vesicular breath sounds of reduced intensity were heard in all areas of auscultation associated with inspiratory rhonchi.

**Figure 1:**
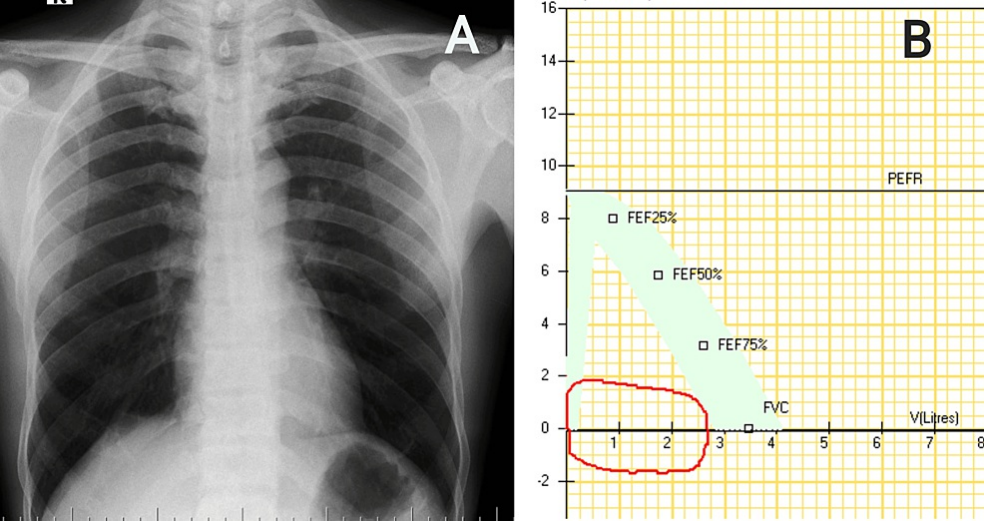
A) Chest radiograph PA view showing hyperinflated lung fields. B) Spirometry revealed flattening of the inspiratory and expiratory graph, suggestive of fixed airway obstruction with forced expiratory time of 1.8 seconds PA, posterior-anterior

On further evaluation, a posterior-anterior (PA) view chest radiograph revealed hyperinflated lung fields, with normal mediastinum (Figure 1A). Spirometry was suggestive of fixed upper airway obstruction (Figure 1B). Routine blood investigations were within normal limits.

**Figure 2:**
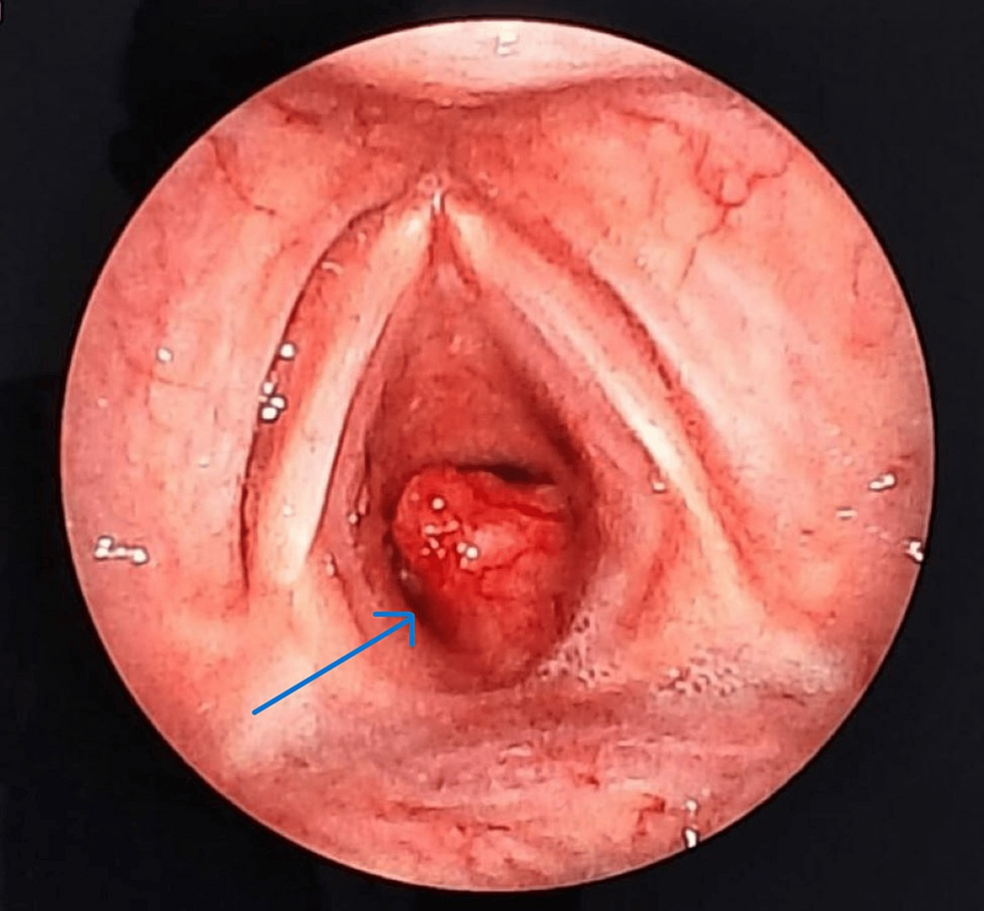
Check bronchoscopy revealed pedunculated mass arising from the right posterolateral subglottic area causing near-total airway obstruction

The patient underwent a check bronchoscopy for evaluation of upper airway obstruction, which revealed a pedunculated mass arising from the right posterolateral subglottic area causing near-total airway obstruction (Figure 2).

**Figure 3:**
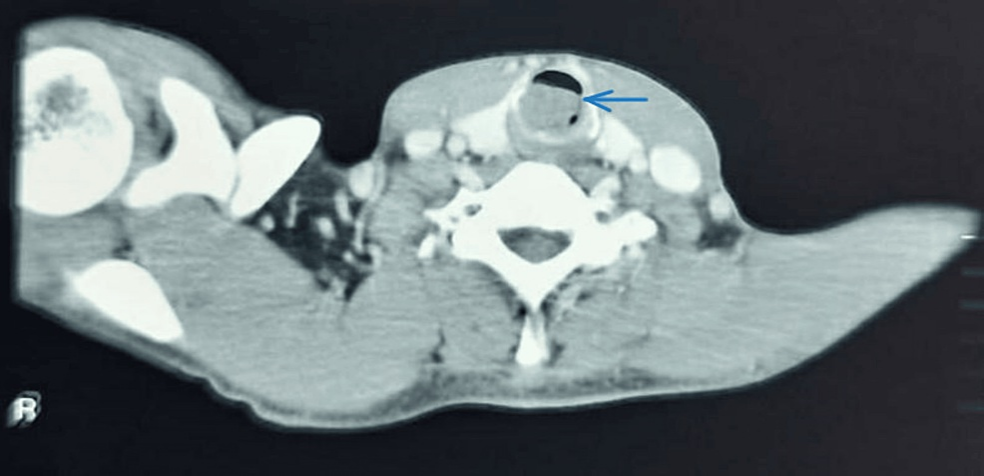
CECT revealing a round to oval well-defined soft tissue density mass lesion arising from the posterolateral aspect of the trachea in the subglottis region with posterior extension CECT, contrast-enhanced computed tomography

A contrast-enhanced CT of the neck and thorax revealed a round to oval well-defined soft tissue density mass lesion arising from the posterolateral aspect of the trachea in the region of subglottis with posterior extension causing near-total obstruction of the tracheal lumen. The study did not reveal any significant involvement of mediastinal or cervical lymph nodes. The tumor mass showed no infiltration into surrounding structures, vessels, or nerves (Figure 3).

**Figure 4:**
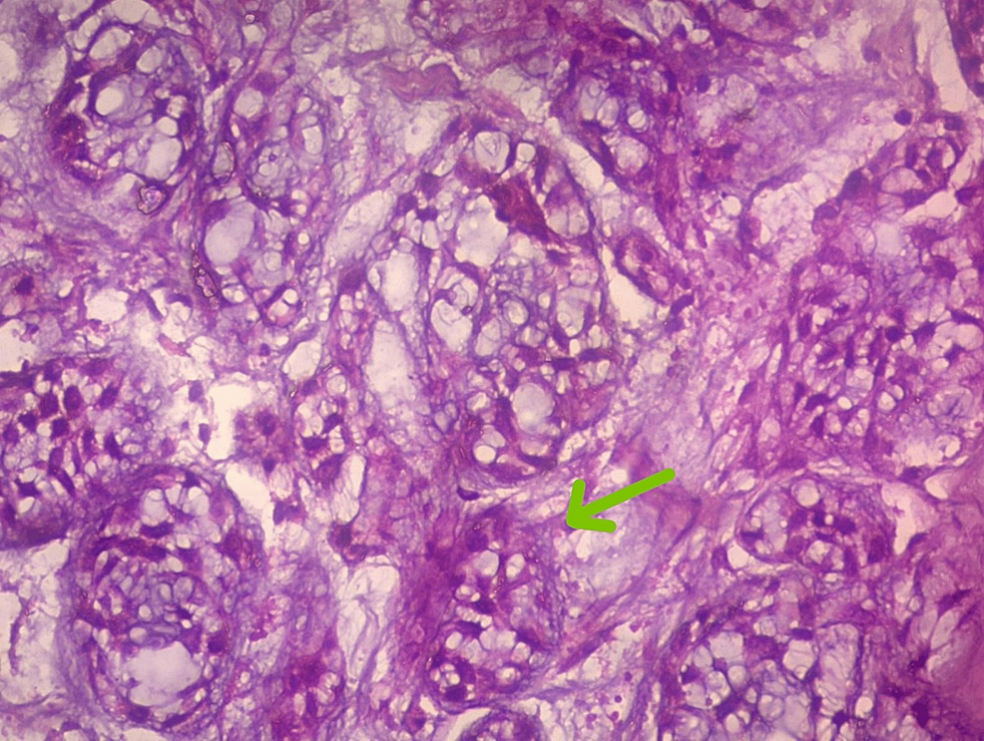
Basaloid cells arranged in a cribriform pattern (40X)

**Figure 5:**
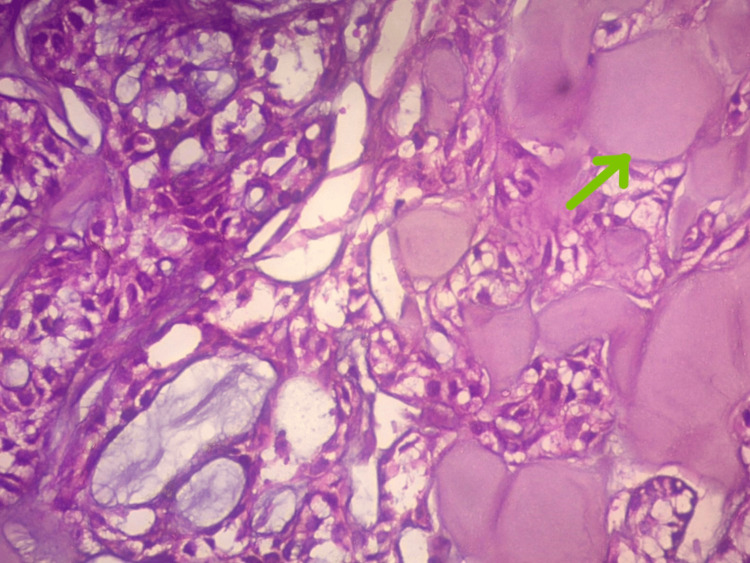
Lobules with abundant hyaline globules (40X)

The patient underwent elective tracheostomy followed by microlaryngeal surgery. Intraoperative findings revealed edematous left aryepiglottic fold, edematous false cords, and fibrous subglottic stenosis. A biopsy was taken. Grossly, the specimen was described as a gray-white soft tissue. Microscopy revealed basaloid cells arranged in a cribriform pattern (Figure 4) and lobules with abundant hyaline globules (Figure 5), with focal areas showing necrosis and hemorrhage. Histopathological examination was suggestive of ACC. Immunohistochemistry of ACC cells revealed positive for S100 and negative for P63, which suggested that the tumor consisted of myoepithelial cells. 

Based on histopathology and CECT head and neck, the patient was diagnosed with carcinoma subglottis cT1 N0 M0 stage I (AJCC) ACC. The patient was advised for laryngectomy with modified radical neck dissection but the patient was unwilling for surgery, hence the patient was treated with radical radiotherapy (RT) via IMRT with neck irradiation. The primary tumor was irradiated with 70 Gy in 35 fractions, 2 Gy/fraction, and 5 fractions/week. During follow-up after six weeks post-RT, the patient had improved clinically.

## Discussion

ACC is a rare malignancy of the larynx, with a mean age of presentation between the fifth and sixth decade of life, lacking a distinct gender predominance [8-10]. The youngest age of ACC presentation is 12 years as reported by Javadi et al. [10]. ACC originates from mixed seromucinous glands of the larynx. Although these glands diminish in number from supraglottis to subglottis, subglottis remains the most commonly affected location. Nearly two-thirds of ACC originates from the subglottic region, and the remaining one-third of cases arise from the supraglottic region, leaving the glottic region an extremely uncommon site for ACC [8,9]. The etiology of ACC is still unclear. ACC of the larynx is a highly invasive and slow-growing tumor with perineural extension with an increased rate of recurrence after initial treatment [10].

The initial presentation of laryngeal ACC varies with the anatomical site and size of the tumor. Tumors arising from supraglottis present with dysphagia and pharyngeal paresthesia. Hoarseness of voice or dyspnea indicates involvement of the glottis. The presence of airway obstruction and stridor implicates the presence of a subglottic tumor [11,12]. Laryngeal ACC remains asymptomatic in the beginning stages, with submucosal non-ulcerated mass, leading to a delay in the diagnosis giving a chance for ACC to spread deeply before a diagnosis is made [11,13]. In this case, the growth was arising from the subglottic area obliterating the trachea and causing dyspnea associated with stridor.

Despite the rarity of cervical lymph node metastasis in ACC accounting for nearly 10-15% of head and neck cases, nodal metastasis poses as one of the commonest reasons for therapy failure [10,14]. However, neck dissection is performed in all the patients with ACC with evidence of nodal metastasis evidenced by physical examination and imaging modalities [14].

ACC has demonstrated a high rate of perineural invasion even in the early stages of the tumor leading to treatment failure and early recurrence. Distant metastasis can occur even in the absence of local recurrence. The most frequent location of distant metastases in ACC is the lungs, with vertebral metastasis contributing to poor survival [10,12]. However, few authors have described lesser survival in tumors originating from minor salivary glands suspecting that they might have originated secondary to an increased rate of distant metastasis [12].

Histopathological evaluation is necessary as the clinical presentation of ACC mimics laryngeal SCC. Based on histology, the World Health Organization classification of tumors, there are three subtypes of ACC namely cribriform, tubular, and solid. The tubular subtype (well differentiated/grade I) has the best prognosis, cribriform (moderately differentiated/grade II) is the commonest variety, and the least common subtype, which is the solid variant (poorly differentiated/grade III), exhibits the worst prognosis. The cells in each of these categories have a mucinous or hyaline matrix. Although basaloid SCC is a well-known entity in the larynx and the prognosis for ACC is significantly better than that of basaloid SCC, it is still necessary to distinguish the basaloid variant of SCC from ACC despite the etiological and histological distinctions between laryngeal SCC and ACC [15]. Often these ACCs demonstrate mixed histological growth patterns hence classified according to the predominant pattern.

Adequate preoperative workup for staging is necessary. Contrast-enhanced computed tomography (CECT) scan of the head and neck is the preferred imaging modality in ACC to assess the location of the primary tumor, extra luminal extension, regional and distant metastasis, and to stage the primary tumor. In our case, the CECT neck and thorax showed a submucosal mass in the subglottic area without any extension to the extra laryngeal soft tissues or regional metastasis, correlating with the histopathological findings. MRI is the modality of choice to evaluate soft tissue and perineural spread of tumors with a sensitivity of 95% [16].

Management of ACC is still a controversy. The primary modality in the treatment of ACC is radical surgery with neck dissection. Post-operative radiotherapy is advised in cases with residual lesions or involved margins. Patients will have benefit in terms of locoregional control. Postoperatively, a dose of 60 Gy-66 Gy can be given based on the presence of positive margin/perineural invasion [17]. Neck node stations can be irradiated based on lymph node involvement as per standard protocol. In settings where RT is used definitely, gross tumors and nerves with tumor involvement can be irradiated to a dose of 70 Gy with irradiation of the neck to standard dose and elective volumes using the same principles of rest of head and neck tumors. Overall survival benefit is 26.5 to 87% and locoregional recurrence rate is 24 to 57% with definitive radiotherapy [18]. The role of RT with concurrent chemotherapy in ACC is ongoing [19].

## Conclusions

ACC of the larynx is a rare and invasive tumor with varied clinical presentations. Accurate diagnosis is crucial for appropriate treatment planning. While surgery is the primary treatment, radiation therapy plays a significant role, especially in cases of residual disease or involvement of margins. Further research is needed to better understand this rare malignancy and optimize treatment strategies.
